# Similar cisplatin sensitivity of HPV-positive and -negative HNSCC cell lines

**DOI:** 10.18632/oncotarget.9028

**Published:** 2016-04-26

**Authors:** Chia-Jung Busch, Benjamin Becker, Malte Kriegs, Fruzsina Gatzemeier, Katharina Krüger, Nikolaus Möckelmann, Gerhard Fritz, Cordula Petersen, Rainald Knecht, Kai Rothkamm, Thorsten Rieckmann

**Affiliations:** ^1^ Department of Otolaryngology and Head and Neck Surgery, University Medical Center Hamburg Eppendorf, Hamburg, Germany; ^2^ Laboratory of Radiobiology and Experimental Radiooncology, University Medical Center Hamburg Eppendorf, Hamburg, Germany; ^3^ Institute of Toxicology, University Medical Center Düsseldorf, Düsseldorf, Germany; ^4^ Department of Radiotherapy and Radiation Oncology, University Medical Center Hamburg Eppendorf, Hamburg, Germany

**Keywords:** HPV, HNSCC, cisplatin

## Abstract

Patients with HPV-positive head and neck squamous cell carcinoma (HNSCC) show better survival rates than those with HPV-negative HNSCC. While an enhanced radiosensitivity of HPV-positive tumors is clearly evident from single modality treatment, cisplatin is never administered as monotherapy and therefore its contribution to the enhanced cure rates of HPV-positive HNSCC is not known. Both cisplatin and radiotherapy can cause severe irreversible side effects and therefore various clinical studies are currently testing deintensified regimes for patients with HPV-positive HNSCC. One strategy is to omit cisplatin-based chemotherapy or replace it by less toxic treatments but the risk assessment of these approaches remains difficult. In this study we have compared the cytotoxic effects of cisplatin in a panel of HPV-positive and -negative HNSCC cell lines alone and when combined with radiation.

While cisplatin-treated HPV-positive strains showed a slightly stronger inhibition of proliferation, there was no difference regarding colony formation. Cellular responses to the drug, namely cell cycle distribution, apoptosis and γH2AX-induction did not differ between the two entities but assessment of cisplatin-DNA-adducts suggests differences regarding the mechanisms that determine cisplatin sensitivity. Combining cisplatin with radiation, we generally observed an additive but only in a minority of strains from both entities a clear synergistic effect on colony formation. In summary, HPV-positive and -negative HNSCC cells were equally sensitive to cisplatin. Therefore replacing cisplatin may be feasible but the substituting agent should be of similar efficacy in order not to jeopardize the high cure rates for HPV-positive HNSCC.

## INTRODUCTION

With an annual incidence of approximately 600.000 new cases worldwide head and neck squamous cell carcinoma (HNSCC) represents the 6^th^ most common cancer type [1, 2]. Major risk factors are the heavy consumption of tobacco and alcohol or infections with human papillomavirus (HPV). HPV-related tumors are preferentially located at the lingual and palatine tonsils in the oropharynx and, in contrast to non-HPV-driven HNSCC, they show high expression of the CDK4/6-inhibitor p16. The incidence of such HPV/p16-positive (HPV(+)) HNSCC is increasing in many countries [3]. The standard treatment of advanced HNSCC regardless of HPV-status is highly intense cisplatin-based radiochemotherapy (RCT), either in the primary setting or as adjuvant therapy after radical surgery. Patients with HPV(+) HNSCC show remarkably better survival rates [4] and various studies are currently testing deintensified therapeutic regimes in order to reduce severe and irreversible side effects for cancer survivors [5, 6]. Deintensification can be achieved by various approaches, such as the reduction of radiation dose or by omitting or exchanging cisplatin-based chemotherapy. Such a replacement of cisplatin by the anti-EGFR-antibody cetuximab is currently being tested in three similar phase 3 trials (De-ESCALaTE, RTOG1016, TROG12.01).

With regard to the mechanisms underlying the favorable prognosis conferred by HPV-positivity, an enhanced tumor radiosensitivity is clearly evident from single modality radiotherapy (RT) treatment [7, 8]. This enhanced sensitivity was also demonstrated on the cellular level when comparing HPV(+) and HPV(−) HNSCC cell lines [9–12]. Since cisplatin is never administered in monotherapy and since only few preclinical data exist for HPV(+) HNSCC [13], it remains unclear whether HPV(+) tumors are also characterized by an enhanced cisplatin-sensitivity. In case of an extraordinary sensitivity and considerable contribution of cisplatin to the high cure rates under standard RCT, omitting or replacing cisplatin may pose the risk of poorer therapeutic efficacy and reduced long-term survival. In this project we therefore compared the cellular responses to cisplatin as well as the cytotoxicity conferred by cisplatin alone and when combined with radiation in a panel of six HPV(+) and five HPV(−) HNSCC cell lines.

## RESULTS

### Cell proliferation

**Figure 1:**
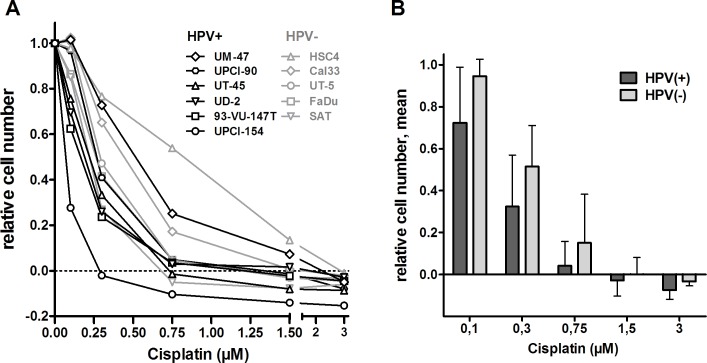
Effect of cisplatin on cell proliferation **A.** Dose response curves. Cells were incubated with the indicated concentrations of cisplatin and incubated for 5 days. Cell numbers were assessed, the numbers of cells seeded was subtracted and the resulting numbers of cells were normalized to the untreated controls. **B.** Mean ± SD of the panels of HPV(+) and HPV(−) cell lines. Data are taken from (A).

In order to evaluate the sensitivity of HPV(+) and HPV(−) HNSCC cells towards cisplatin, we treated HNSCC cell lines with increasing doses of the drug for 5 days and assessed the resulting number of cells. We observed a relatively high variation between the individual strains ranging from 100% to only 23% growth inhibition at a concentration of 0.3μM cisplatin (Figure 1A). At all concentrations the group of HPV(+) HNSCC strains demonstrate a trend towards more pronounced proliferation inhibition and toxicity (Figure 1B) but statistical significance was not reached (*p* = 0.165).

### Cellular responses and DNA-adducts

To assess whether there are principal differences in the cellular responses of HPV(+) and HPV(−) HNSCC cells to cisplatin, cells were treated with a concentration of 1μM (0.3μg/ml). This concentration is in the lower range of the total cisplatin plasma concentrations observed after the initial fast decline a few hours after infusion [14] and therefore represents a physiologically relevant dose. We assessed the cell cycle response, the induction of apoptosis, the DNA damage marker γH2AX and the formation and maintenance of cisplatin-DNA-adducts in pairs of HPV(+) and HPV(−) cell lines with similar sensitivity. To this end we chose HSC4 and UM-SCC-47, which still demonstrated proliferation at 1μM cisplatin, as resistant cell lines, FaDu and UD-SCC-2, which demonstrated a steady state in cell number, as intermediately sensitive strains and SAT and UPCI-SCC-154, which showed a decrease in cell number, as sensitive strains (see Figure 1A).

### Cell cycle

**Figure 2:**
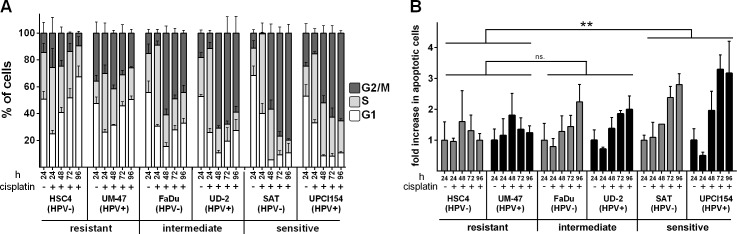
Cell cycle and apoptosis **A.** Cell cycle: Cells were incubated with 1μM cisplatin. After the times indicated the cells were harvested, fixed and the cell cycle distribution was assessed using propidium iodide staining. **B.** Apoptosis: Cells were treated as in (A) but harvested and subjected to flow cytometric assessment of caspase activity. The fractions of caspase positive cells in untreated samples were set as 1. In (B) statistically significant differences between groups are indicated by asterisks (*p* = 0.0021). Significance was reached at 72h (****) and 96h (****) (RM two-way ANOVA with post-hoc analyses (Holm-Sidak)).

As cisplatin-DNA-adducts are obstacles for DNA replication fork progression, cells accumulate in the S-phase of the cell cycle upon cisplatin exposure. Depending on the dose and on the ability to repair and bypass the acquired lesions, cells slowly progress through the S- and then an often prolonged G2-phase towards mitosis. In line with the sensitivity as observed in the proliferation assay, we observed an initial accumulation of cells in the S-phase which in both sensitive cell lines was followed by a constant increase of cells arrested in G2 (Figure 2A). In contrast, the resistant strains HSC4 and UM-SCC-47 showed less accumulation in the S-phase followed by a complete recovery of the cell cycle distribution. Intermediately sensitive cells showed an initial accumulation in the S- and G2-phase, similar to the sensitive strains, but at later time points the portion of cells in the G2-phase declined. Notably, we did not observe any principal differences between HPV(+) and HPV(−) cell lines.

### Apoptosis

The induction of apoptosis upon cisplatin exposure is believed to be an important mediator of cell death and inactivation [15]. To determine to what extent the cell line specific accumulation of cells in the S- and G2-phases was accompanied by the induction of apoptosis, we assessed caspase activation upon treatment with 1μM cisplatin. In the resistant cell lines we observed an early peak of cells showing caspase activation that was followed by a fast decline to baseline levels (Figure 2B). In contrast, sensitive cells showed a steady increase in the portion of apoptotic cells that was also observed in cells of intermediate sensitivity but to a lesser extent. In absolute numbers however, the percentages of cells demonstrating caspase activation upon cisplatin treatment remained below 10%, except for the UD-SCC-2 strain, which contained a profound number of cells with caspase activity already without treatment (Suppl. Figure 1). In conclusion, these low numbers call into question whether apoptosis plays a major role in the sensitivity towards cisplatin under our experimental conditions.

### γH2AX

**Figure 3:**
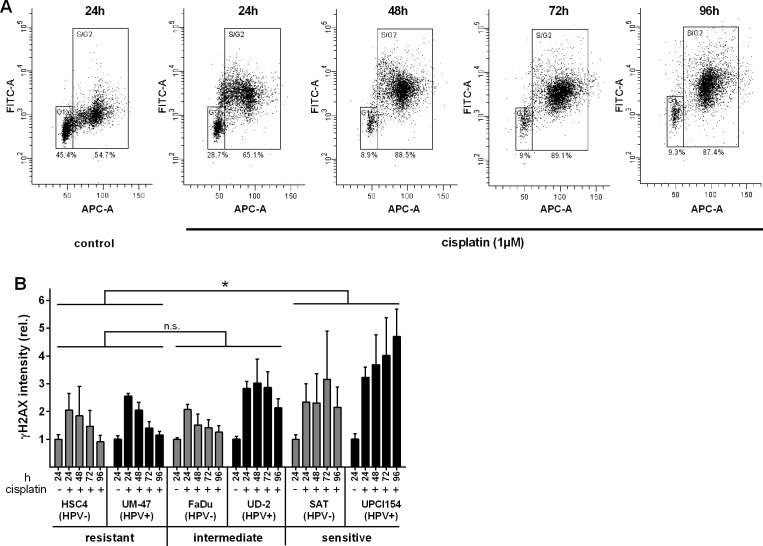
Histone 2AX phosphorylation **A.** Example of flow cytometric assessment of the γH2AX level in relation to the cell cycle phase/DNA content in UPCI-SCC-154. Cells were incubated with 1μM cisplatin. After the times indicated the cells were harvested, fixed, stained for γH2AX (FITC-A) and counterstained for DNA content (APC-A). Numbers below the gates depict the percentage of cells in the respective cell cycle phases. **B.** Quantification. Graphs represent the fold change of γH2AX staining intensity of the respective S/G2/M-phase cells (gate “S/G2”) relative to the staining of the corresponding untreated S/G2/M-phase cells. Statistically significant differences between groups are indicated by asterisks (*p* = 0.0117). Significance was reached at 72h (***) and 96h (****) (RM two-way ANOVA with post-hoc analyses (Holm-Sidak)).

γH2AX is the phosphorylated form of histone 2AX. H2AX is phosphorylated upon various forms of DNA-damage and γH2AX is a marker of DNA double-strand breaks and stalled replication. In contrast to the distinct foci observed in immunofluorescence analyses upon ionizing irradiation [11, 16], cisplatin exposure more often led to a pan-nuclear staining especially of S-phase cells likely due to the stalling of multiple replication forks (not shown). Therefore, we performed flow cytometric analyses to quantify cellular γH2AX levels (Figure 3A). We again observed marked differences between resistant and sensitive cells. While we observed an initial induction of H2AX-phosphorylation in the S&G2 phase in all cell lines, γH2AX declined over time in resistant cells (Figure 3B). In contrast, the γH2AX levels remained high in both sensitive strains. The two cell lines of intermediate sensitivity showed a divergent response with that of FaDu resembling the phenotype of the resistant cells and that of UD-SCC-2 resembling the phenotype of the sensitive cells.

### Cisplatin-DNA-adducts

**Figure 4:**
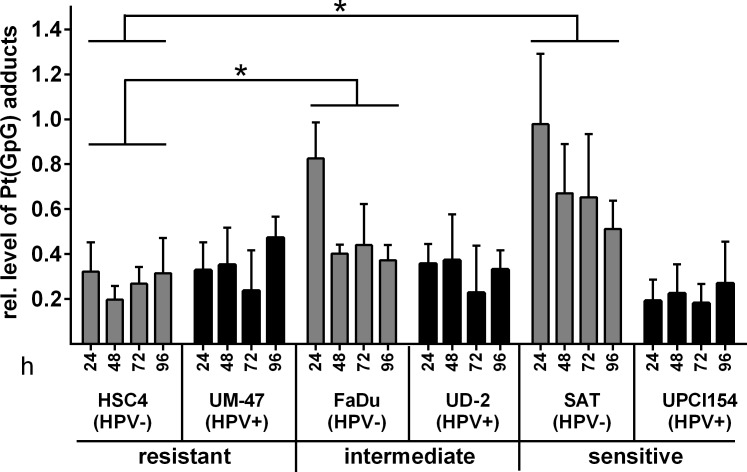
Cisplatin-DNA-adduct levels Cells were incubated with 1μM cisplatin. After the times indicated the cells were harvested and subjected to genomic DNA isolation. Pt(GpG) adduct levels were determined using Southwestern blots and staining with an anti-Pt(GpG)-antibody. Methylenblue staining of whole genomic DNA was performed as a loading control. Statistically significant differences between groups are indicated by asterisks (HSC4 vs. FaDu: *p* = 0.0232 and HSC4 vs. SAT: *p* = 0.300). In both evaluations significance was reached at 24h (**** and ***) and for HSC4 vs. SAT also at 48h and 72h (* and *) (RM two-way ANOVA with post-hoc analyses (Holm-Sidak)).

Cisplatin exerts its cytotoxic effects largely *via* the induction of cisplatin-DNA inter- and intrastrand crosslinks. The latter are far more frequent and can be detected by adduct-specific antibodies [17]. We assessed the level of cisplatin-guanin-guanin intrastrand crosslinks (Pt(GpG)) at different time points after treatment with 1μM cisplatin using a Southwestern slot blot. In contrast to the cellular responses described above, the outcome was only partly associated with the sensitivity of the cell lines. Both resistant strains showed only weak adduct formation while the HPV(−) strains FaDu and SAT demonstrated a high initial adduct level at 24h after cisplatin addition which declined thereafter but especially in the highly sensitive SAT cells remained at a fairly high level until the end of the time course (Figure 4). The HPV(+) strains UD-SCC-2 and UPCI-SCC-154 cells, however, showed DNA-adduct levels comparable to the resistant strains. In fact, the most sensitive strain, UPCI-SCC-154, showed the lowest adduct level of all cell lines.

So, while none of the assays reflecting the cellular responses towards cisplatin (cell cycle distribution, apoptosis and γH2AX formation) demonstrated a general difference between HPV(+) and HPV(−) cell lines, the cisplatin-DNA-adduct assessment suggests that cisplatin sensitivity may be governed by different mechanisms with cisplatin-adduct excision from DNA possibly being a more critical step in HPV(−) HNSCC.

### Colony formation

**Figure 5:**
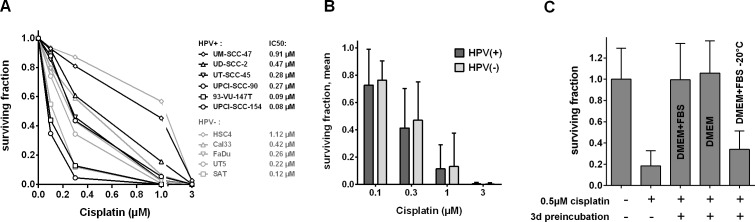
Effect of cisplatin on colony formation **A.** Dose response curves. Cells were seeded in defined numbers at low density to allow colony formation. Cisplatin was added after three hours and cells were incubated for 1 week before media exchange and incubation until formation of colonies. Standard deviations were omitted for clarity, IC50 values of all cell lines are presented. **B.** Mean and SD of the panels of HPV(+) and HPV(−) cell lines. Data are taken from (A). **C.** Decrease of biological activity. Cisplatin was preincubated under different conditions for three days and assessed for the ability to inhibit colony formation of UT-SCC-5 cells. Cisplatin was added three hours after seeding and cells were incubated for 24 h before media exchange and incubation until formation of colonies. All data in A, B and C were normalized to the respective untreated controls.

In addition to the effects observed on proliferation, we also assessed the ability of all HPV(+) and HPV(−) cell lines to form colonies upon cisplatin treatment as a more robust readout for cytotoxicity. Here, we observed an even more pronounced variation among the different strains (Figure 5A). While those cell lines characterized as most resistant or highly sensitive in the proliferation assay showed a similar phenotype in the colony formation assay, some variations were observed for other strains and, importantly, the trend towards a higher sensitivity of HPV(+) strains was lost (Figure 5B).

In these colony formation assays we used an experimental setup with a long incubation period of 1 week, to compare the long-term effects of relatively low doses of cisplatin instead of using short term pulses with high concentrations that are unlikely to be reached in patients’ tumor tissue. Testing a more common but still relatively long incubation time of 24h in four selected strains, we observed a similar sequence of sensitivities (Suppl. Figure 2). However, when considering the profound decrease in incubation time, the cytotoxicity conferred by 24h of cisplatin treatment was found to be surprisingly similar to the results obtained after 1 week of treatment. Furthermore, the rapid recovery of the resistant HSC4 and UM-SCC-47 cells from cell cycle arrest, apoptosis-induction and γH2AX formation, which was observed in the presence of cisplatin (Figures 2, 3, 4), prompted us to investigate the biological activity of the compound after incubation under cell culture conditions. Surprisingly, after three days of preincubation, cisplatin completely failed to inhibit colony formation (Figure 5C). Addition of FBS was not necessary for inactivation whereas storage at −20°C largely preserved cytotoxicity. This loss of biological activity likely explains the recovery from the various cellular stress responses observed in the resistant HSC4 and UM-SCC-47 cells.

### Interaction with radiation

**Figure 6:**
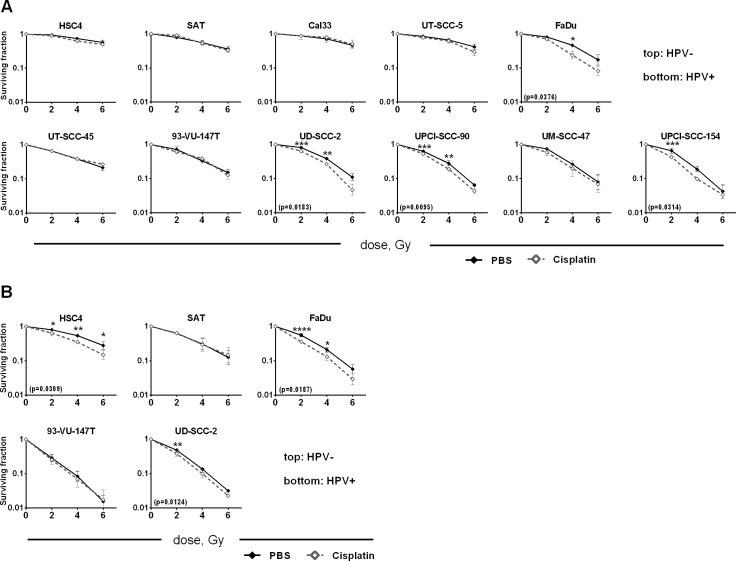
Cisplatin and radiation Cells were seeded in defined numbers at low density to allow colony formation. **A.** Cisplatin (IC50 of each individual strain) was added after 3 hours. 72 h after cisplatin addition the cells were irradiated with the doses indicated. After a total of 1 week of cisplatin incubation the medium was exchanged and the cells were incubated until formation of colonies. **B.** Higher cisplatin dose and earlier irradiation: Cells were treated as in (A) except for the addition of a higher concentration of cisplatin (IC75 of each individual strain) and earlier irradiation (6h after addition of cisplatin). Colony counts were normalized to the respective non-irradiated sample to show only synergistic effects. In case of statistically significant differences the p-value is given in brackets and individual statistically different dose points are indicated by asterisks (RM two-way ANOVA with post-hoc analyses (Holm-Sidak)).

In the curative treatment of HNSCC cisplatin is combined with radiotherapy and in the field of radiation oncology it is often referred to as a potent radiosensitizer, meaning that beyond its single agent cytotoxicity it also enhances the effects of radiation. Therefore we also assessed the effect of combined treatment in the colony formation assay. Clinically, radiotherapy is administered in daily fractions while cisplatin can be administered in multiple ways varying typically from three times 100mg/m^2^ administered every three weeks to 30mg/m^2^ weekly. In any case most fractions of radiotherapy are not given at the time of cisplatin administration. We therefore chose to irradiate three days after cisplatin supplementation, a time point at which cisplatin is no longer active in the medium (Figure 5C) but various cellular responses are still observable in most cell lines (Figures 2, 3, 4). To account for the different sensitivities of the individual strains, we administered the individual inactivating concentration 50% (IC50) as assessed in the colony formation assay (Figure 5A, Suppl. Figure 3). Colony formation of all cell lines was reduced upon cisplatin treatment (Suppl. Figure 4). To assess the interaction of cisplatin and irradiation the cytotoxic effect of cisplatin was subtracted by normalizing the data to the respective non-irradiated samples. We observed a statistically significant reduction in cell survival in four cell lines, namely UD-SCC-2, UPCI-SCC-90, UPCI-SCC-154 cells (HPV+) and FaDu (HPV-) (Figure 6A). Using a higher cisplatin concentration (IC75, Suppl. Figure 3) and an earlier irradiation time point of 6h after cisplatin administration in some cell lines, we obtained partly the same and partly divergent results, demonstrating some variability and dependence on the experimental setup (Figure 6B). In summary, these data argue against a robust effect of cisplatin in enhancing the efficiency of ionizing radiation in either of the two entities.

## DISCUSSION

In this project we asked whether HPV(+) HNSCC cells are more sensitive towards cisplatin than HPV(−). To answer this question we used a panel of five HPV(−) and six HPV(+) strains. To the best of our knowledge this HPV(+) panel contains all cell lines derived from primary, untreated HPV(+) HNSCC that have been described in the literature. Proliferation was found to be somewhat more severely inhibited in HPV(+) strains (Figure 1) but this was not accompanied by a generally enhanced cytotoxicity as assessed by the ability to form colonies under cisplatin treatment (Figure 5A, 5B). In both panels the sensitivity towards cisplatin shows huge variations. This is likely to represent the situation in the clinic and so far no predictive biomarker for tumor sensitivity towards cisplatin has been established for routine clinical use. This variation in cisplatin sensitivity also demonstrates the necessity to use large cohorts of cell lines because a comparison of e.g. 2 HPV(+) vs. 2 HPV(−) strains would simply yield random results. Using a number of different approaches, we did not observe any differences in the cellular responses, namely cell cycle distribution, apoptosis induction and γH2AX formation, upon cisplatin treatment in matched pairs of HPV(+) and HPV(−) cell lines with similar sensitivity (Figures 2, 3). In contrast we had previously observed a clearly distinct cell cycle response after ionizing irradiation also in cell lines of similar radiosensitivity [11]. In a study using 19 HNSCC cell lines Martens-de Kemp et al. found the cisplatin-DNA-adduct level to be the only predictor of cisplatin sensitivity while the expression level of influx and efflux transporters or DNA repair proteins showed no association [18]. In that study the adduct level was only determined in HPV-negative cell lines. In our study, despite differences in cisplatin concentration and timing, the observed DNA-adduct levels of the three HPV(−) strains are well in line with that observation. In contrast all three HPV(+) cell lines showed comparably low adduct levels, even the highly cisplatin sensitive UPCI-SCC-154 cells. This suggests effective removal of cisplatin from the primary lesions by the nucleotide excision repair (NER) pathway but a deficiency in the processing of the resulting repair intermediates. A similar phenotype was previously observed in cisplatin hypersensitive CL-V5B cells [19]. UPCI-SCC-154 cells were recently described to be defective in homologous recombination repair (HRR) due to the high expression of p16 and/or the inability to form Rad51 foci [20, 21]. HRR is required for repair steps after cisplatin excision, especially in the repair of interstrand crosslinks in the S and G2-phase [22]. Whilst an HRR defect could therefore explain the sensitivity of UPCI-SCC-154, cisplatin resistant UM-SCC-47 cells were described to be equally HRR deficient [21] and also express similar levels of p16 [11]. Therefore additional research into the underlying mechanisms is required to fully explain these interesting findings.

In contrast to many other studies we had chosen experimental setups with long cisplatin incubation periods to be able to work with physiologically low concentrations [14, 23]. Cisplatin is stable in aqueous solutions containing at least 0.45% NaCl (77mM) and is routinely delivered and stored in 0.9% NaCl (154mM) at room temperature. After cellular uptake, due to the low intracellular chloride concentration the cisplatin-bound chloride ligands are replaced by water, which generates the active forms of cisplatin [15]. The chloride concentration of the cell culture media used here is around 120mM, very similar to blood and interstitial body fluid. Therefore it was unexpected for us to see that after 3 days of incubation cisplatin no longer demonstrated any cytotoxicity. Nevertheless, in sensitive cell lines we observed cellular responses increasing even beyond 72h (Figures 2, 3). It is tempting to speculate that in patients the same phenomenon may occur and contribute to rapid detoxification in addition to renal elimination and consequently also to treatment failure of resistant tumors. Further research is necessary to clarify these issues.

RT is normally delivered in daily fractions of 2 Gy over about 6 weeks and only very few fractions are administered synchronously with the addition of cisplatin. We therefore chose a time point of three days after cisplatin administration where marked differences in the cellular cisplatin responses were evident between cell lines of different cisplatin sensitivity. Using this setup cell survival will depend on both the resulting cellular radiosensitivity and on cell proliferation between seeding and irradiation. As observed before [11] HPV(+) strains were more radiosensitive than HPV(−). Using the individual IC50 of cisplatin for each cell line we observed only moderate synergistic effects and, importantly, only in a minority of strains (Figure 6A). Using a higher concentration (IC75) and an earlier time point of 6h after cisplatin administration we observed partly different results (Figure 6B). Cisplatin resistant HSC4 cells were sensitized only at the earlier time point, which can be easily explained by the marked recovery of these cells after 72h (Figure 2A, 2B). The sensitive strains SAT and 93-VU-147T, however, were not sensitized under any of these conditions and, unexpectedly, the intermediately sensitive UD-SCC-2 cells demonstrated a higher reduction in cell survival with the lower concentration at the later time point (1.74 fold averaged over all three dose points) as compared to treatment with the higher concentration at the earlier time point (1.38 fold averaged over all three dose points). These results show that on the cellular level the enhancement of radiation sensitivity of HNSCC tumor cells by cisplatin is diverse and can depend on the experimental setup, which may explain the differences between our and another recent study [13]. Our results call into question whether cisplatin - besides its cytotoxic effects - can efficiently sensitize HNSCC tumors towards radiation. However, it needs to be considered that *in vivo* additional mechanisms, such as the inhibition of tumor cell repopulation during the course of therapy may also play a role. After all, the benefit in five-year overall survival that cisplatin adds to radiotherapy in HNSCC patients is in the range of 10% [24, 25], which is well in line with a reasonable number of tumors showing considerable resistance against cisplatin mediated cytotoxicity and enhancement of radiation induced cell killing.

Currently three similar phase 3 trials are testing the replacement of cisplatin by cetuximab in combination with RT in HPV(+) oropharyngeal squamous cell carcinomas(OPSCC) with curative intent (De-ESCALaTE, RTOG1016, TROG12.01). While our data suggest that HPV(+) tumors may not possess a generally enhanced sensitivity towards cisplatin, they still show that cisplatin can be highly toxic and lead to a further increase in cell kill when combined with irradiation, the latter preferably in HPV(+) strains. Therefore the addition of cetuximab should at least be similarly effective in order not to jeopardise the high cure rates achieved with cisplatin-based RCT. In this context a subgroup analysis of the Bonner trial showed that p16-positive OPSCC had a higher benefit from the addition of cetuximab to irradiation as compared to p16-negative OPSCC [26]. Other trials and data, however, heavily question the use of EGFR inhibition in combination with irradiation and especially chemoirradiation in both HPV(−) and HPV(+) HNSCC [27–31]. Using five of the six HPV(+) cell lines also used here we previously did not find any radiosensitizing effect of cetuximab [32]. In contrast, inhibitors of Chk1 and PARP conferred radiosensitization [32, 33]. Therefore, from a radiobiological perspective we suggest that replacing cisplatin with inhibitors of DNA repair or the DNA damage response may be more promising than the use of EGF-pathway inhibitors and should be investigated further in in-vivo preclinical studies for a potential clinical use in deintensified regimes.

## MATERIALS AND METHODS

### Cells and cell culture

All cell lines were grown in DMEM (Gibco) supplemented with 10% fetal bovine serum (FBS) (BiochromeAG) and 2mM glutamine (Gibco) at 37°C, 10% CO_2_ and 100% humidification. All HPV(+) and HPV(−) cell lines utilized were described previously [11, 34], except UPCI-SCC-90 (= UPCI:SCC:90 ; obtained from the German Collection of Microorganisms and Cell Lines (DSMZ), originally deposited by Prof. S. Gollin, Pittsburgh, USA). All cells were identified by a short tandem repeat multiplex assay (Applied Biosystems). Cisplatin was generally administered to exponentially growing cells 3 h after seeding.

### Cell proliferation and colony formation assay

For cell proliferation analysis, 5 × 10^4^ cells were seeded into T25 cell culture flasks and treated with various doses of cisplatin 3 h later. The numbers of cells were assessed after 5 days and the number of cells initially seeded was subtracted. Results were normalized to the respective untreated control.

Cytotoxicity was determined in a preplating colony formation assay. Briefly, subconfluent cell cultures were seeded in defined numbers into T25 cell culture flasks and treated with cisplatin 3 h later. After 1 week the cells were washed with PBS and incubated in fresh medium without cisplatin. Incubation for colony formation varied between 2 and 6 weeks depending on the doubling time of the respective cell line. Additional irradiation was performed as indicated. Samples treated with cisplatin and/or irradiation were allowed to grow for an extended period of time, as colony formation in some strains was apparently delayed. The number of colonies containing more than 50 cells was assessed. In the case of UM-SCC-47, all samples were seeded with 5000 feeder cells (UM-SCC-47; 20 Gy) per flask to support plating efficiency and for slowly proliferating cells (UPCI-SCC-154, UPCI-SCC-90 and SAT) the medium was changed to Amniomax C-100 medium plus 7,5% Amniomax Supplement (both Gibco) and 7,5% FBS (Biochrom AG) at 4 weeks after seeding to facilitate colony formation.

### Cell cycle assessment

Cells were harvested, fixed with 70% ethanol, briefly washed with PBS/0.2% Triton X-100 and subsequently incubated with 100 ng/ml RNAse A and 10 μg/ml propidium iodide in PBS/0.2% Triton X-100 for 30 min at room temperature in the dark. Flow cytometric analysis was performed using a FACS Canto with FACS Diva Software (Becton Dickinson). The portion of cells in the respective cell cycle phases was calculated using ModFit LT^TM^ software (Verity Software House, Inc.).

### Caspase activity

Detection of caspase activity was performed utilizing the FAM-FLICA™ Poly Caspases Assay Kit (Immunochemistry Technologies) according to the manufacturer's instructions. Flow cytometric analysis was performed on a FACS Canto with FACS Diva Software (Becton Dickinson).

### γH2AX assessment

Cells were harvested, fixed with PBS/4% formaldehyde for 10 min and permeabilized with PBS/0.2% Triton X-100 before blocking for 30 min with PBS/3% BSA /0.2% Triton X-100. The cells were subsequently incubated (1h; RT) with a mouse-anti-γH2AX antibody (clone JBW301, Millipore) in blocking solution, washed four times with PBS/0.1 % Tween20 before incubation (1h; RT) with anti-mouse DyLight488 (Jackson Immunoresearch) and were then washed again four times. DNA counterstaining was performed using FxCycle FarRed (Molecular Probes) plus 100 ng/ml RNAse A and 0,2% Triton X-100 for 30 min at room temperature in the dark. Flow cytometric analysis was performed using a FACS Canto with FACS Diva Software (Becton Dickinson).

### Analysis of cisplatin-induced formation of DNA intrastrand crosslinks

The level of Pt-(GpG) intrastrand crosslinks was determined by Southwestern blot analysis. To this end, cells were treated with cisplatin as indicated and harvested by trypzination. Cell pellets were immediately frozen at −80°C until extraction of genomic DNA using the NucleoSpin Tissue Kit (Macherey Nagel). Concentration and purity of the DNA were determined photometrically, DNA integrity was confirmed using agarose gel electrophoresis and ethidiumbromide staining. 0.5 μg of the genomic DNA was diluted with TE buffer up to 100 μl, denatured (10 min, 95°C) and subsequently cooled on ice before 100 μl ice cold ammonium acetate (2 M) (MERCK) was added. The DNA was transferred onto a nitrocellulose membrane soaked in 1 M ammonium actate by using a vacuum pump. After washing (1 M ammonium acetate and water), the membrane was incubated with 5 x SSC (10 x SSC: 1.5 M NaCl (VWR International), 150 mM sodium citrate (MERCK), pH 7.0) for 5 min and baked for 2 h at 80°C before it was blocked in 5 % non-fat milk in TBS/0.1 % Tween 20 (over night; 4°C). After washing (TBS/0.1 % Tween 20) incubation with the primary antibody directed against Pt-(GpG) intrastrand crosslinks [17] was performed (1 h; RT). After a further washing step peroxidase-conjugated anti-rat IgG secondary antibody was added (2 h; RT). Pt-(GpG) intrastrand crosslinks were detected by chemiluminescence using the Fusion FX7 imaging system. Autoradiographies were densitometrically analyzed with ImageJ 1.48r. To ensure equal loading, the membrane was stained with methylene blue (MP Biomedicals).

### X-irradiation

Cells were irradiated at room temperature with 200 kV X-rays (Gulmay RS225, Gulmay Medical Ltd., 15 mA, 0.8 mm Be + 0.5 mm Cu filtering, dose rate - 1.2Gy/min).

### Data evaluation

Data analysis and statistical evaluation were performed using GraphPad Prism (GraphPad Software, Inc.). All experiments were performed at least three times and values presented are mean ± SD unless noted otherwise. Statistical evaluation was performed using repeated measures (RM) two-way ANOVA test for analysis of whole data sets and post-hoc analyses (Holm-Sidak) to further assess the levels of significance at individual time or dose points.

## SUPPLEMENTARY MATERIAL FIGURES


