# Supplementary material for: Similar cisplatin sensitivity of HPV-positive and -negative HNSCC cell lines
Source: Oncotarget. 2016 Apr 26;7(24):35832–42. doi: 10.18632/oncotarget.9028 (PMC5094966; doi:10.18632/oncotarget.9028)
Supplement: Supplementary file 1 [file oncotarget-07-35832-s001.pdf]

Similar cisplatin sensitivity of HPV-positive and -negative HNSCC cell lines

Supplementary Material

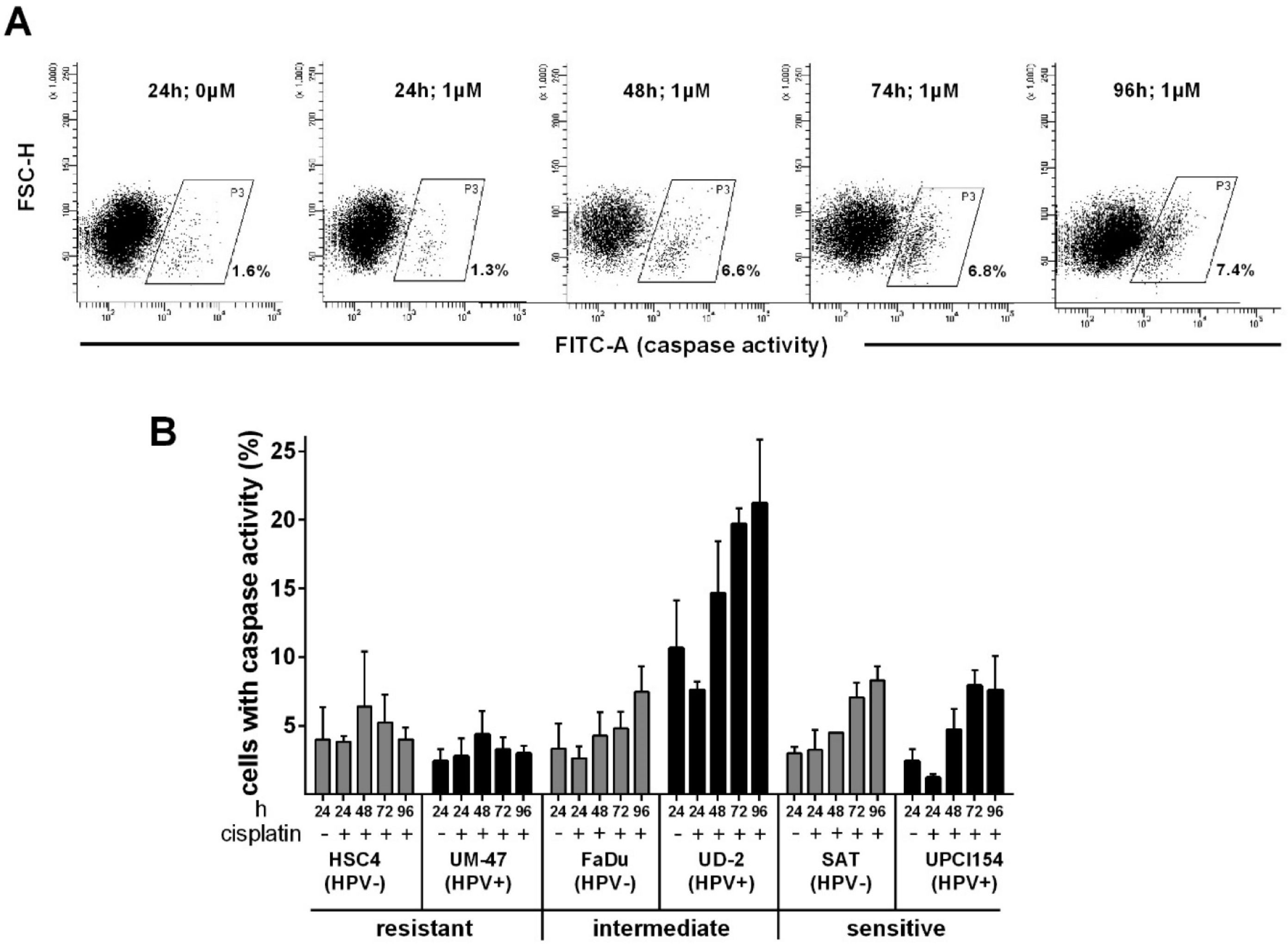

Supplementary Figure 1. Apoptosis induction, absolute values. Cells were incubated with 0 or 1µM cisplatin. After the times indicated the cells were harvested and subjected to flow cytometric assessment of caspase activity. (A) Examples of flow cytometric assessment of apoptosis in UPCI-SCC-154. Numbers depict the percentages of caspase positive cells (gate P3). (B) Graphs represent the absolute levels of cells with caspase activity. Data are based on the same experiments as Fig. 2B.

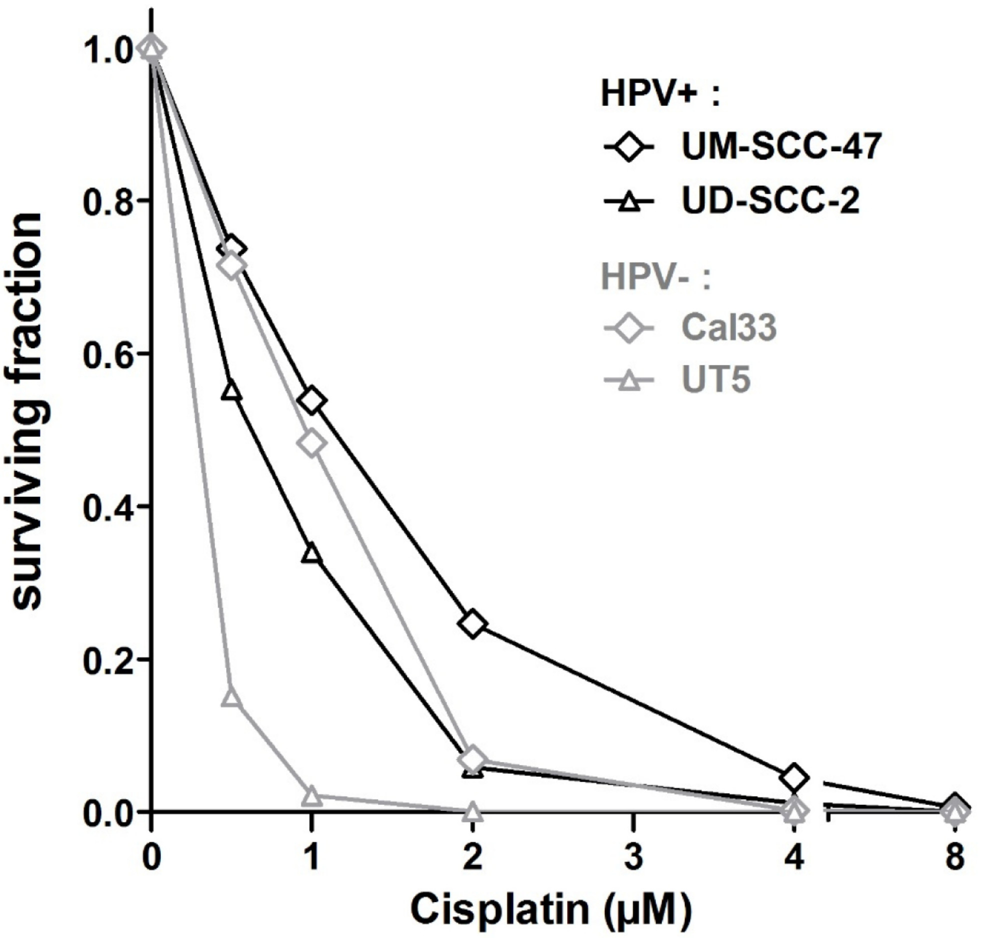

Supplementary Figure 2. Colony formation after 24h cisplatin incubation. Cells were seeded in defined numbers at low density to allow colony formation. Cisplatin was added after three hours and cells were incubated for 24 h before media exchange and incubation until formation of colonies.

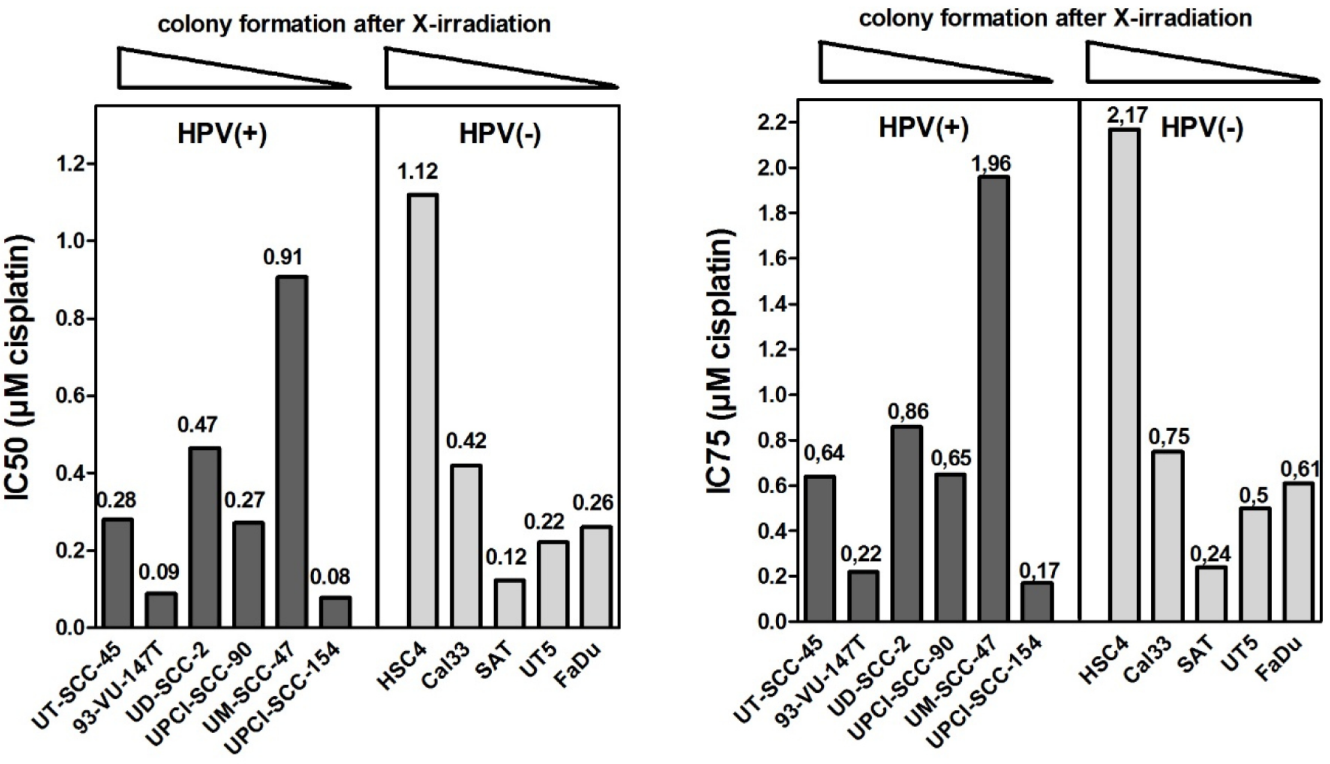

Supplementary Figure 3. Cisplatin IC50 and IC75 values of HPV-positive and -negative HNSCC cell lines. Data are taken from Fig 5A. The depicted order of radiation sensitivity was transferred from previous studies [10,11].

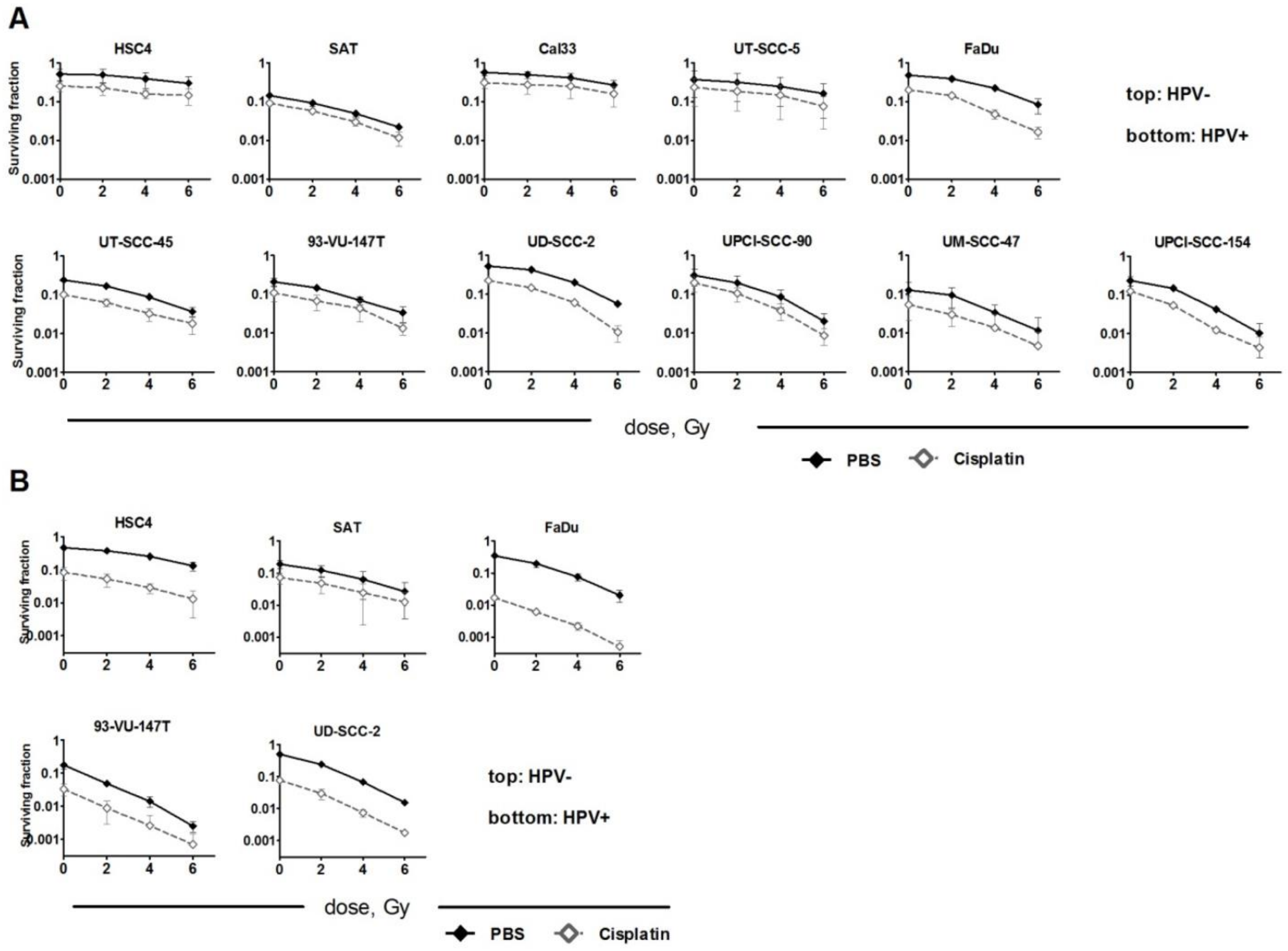

Supplementary Figure 4. Cisplatin and radiation. Data are based on the same experiment as Fig. 6 but values were not normalized to the respective non-irradiated control to also depict additive effects.
